# N95 respirator mask breathing leads to excessive carbon dioxide inhalation and reduced heat transfer in a human nasal cavity

**DOI:** 10.1063/5.0061574

**Published:** 2021-08-23

**Authors:** Hana Salati, Mehrdad Khamooshi, Sara Vahaji, Farid C. Christo, David F. Fletcher, Kiao Inthavong

**Affiliations:** 1 Mechanical and Automotive Engineering, School of Engineering, RMIT University, Bundoora, Australia; 2 School of Engineering, Faculty of Science Engineering and Built Environment, Deakin University, Victoria, Australia; 3 School of Chemical and Biomolecular Engineering, The University of Sydney, NSW 2006 Australia

## Abstract

Face masks and respirators are used to filter inhaled air, which may contain airborne droplets and high particulate matter (PM) concentrations. The respirators act as a barrier to the inhaled and exhaled air, which may change the nasal airflow characteristics and air-conditioning function of the nose. This study aims to investigate the nasal airflow dynamics during respiration with and without an N95 respirator driven by airflow through the nasal cavity to assess the effect of the respirator on breathing conditions during respiration. To achieve the objective of this study, transient computational fluid dynamics simulations have been utilized. The nasal geometry was reconstructed from high-resolution Computed Tomography scans of a healthy 25-year-old female subject. The species transport method was used to analyze the airflow, temperature, carbon dioxide (CO_2_), moisture content (H_2_O), and temperature distribution within the nasal cavity with and without an N95 respirator during eight consecutive respiration cycles with a tidal volume of 500 ml. The results demonstrated that a respirator caused excessive CO_2_ inhalation by approximately 7× greater per breath compared with normal breathing. Furthermore, heat and mass transfer in the nasal cavity was reduced, which influences the perception of nasal patency. It is suggested that wearers of high-efficiency masks that have minimal porosity and low air exchange for CO_2_ regulation should consider the amount of time they wear the mask.

## I. INTRODUCTION

Healthcare workers and medical response teams are recommended to wear personal protective equipment while undertaking healthcare during pandemics. The N95 respirator is a respiratory personal protective equipment that protects against infectious respiratory diseases, including COVID-19. N95 respirators are made of four layers, which causes resistance to inhalation and exhalation airflow. This resistance is expected to affect nasal airflow, where accumulated exhaled carbon dioxide (CO_2_) concentration in the mask region is re-inhaled. The augmentation of CO_2_ in the mask zone could create exposure to increased levels of CO_2_ in subsequent breaths that could cause adverse physiological effects, over prolonged use. This is in contrast to surgical masks, which do not affect relevant physiological changes in gas exchange under prolonged rest or brief walking.^1^

Light-headedness, headache, and high blood pressure^2,3^ are symptoms that have been observed after wearing an N95, which can be associated with shortness of fresh air for inhalation. Elisheva and Rosner^4^ reported adverse side effects, including headaches, rash, acne, skin breakdown, and impaired cognition in the majority of 343 healthcare professionals working in response to COVID-19. These side effects re-iterate past findings of headaches^3^ and adverse skin reactions, such as rashes, acne, and itching from mask use.^5–7^ Rebmann *et al.*^2^ investigated the effect of wearing an N95 on outcome variables for ten nurses using longitudinal analysis based on a multivariate linear regression model, and they concluded that the CO_2_ level increased significantly compared with baseline measures, leading to light-headedness and high blood pressure.

Atangana *et al.*^8^ assessed the relationship between wearing a facemask and CO_2_ inhalation and recommended a full mask respirator due to better air circulation compared with other respirators and facemasks. Mardimae *et al.*^9^ demonstrated that a modified N95 mask could administer clinically equivalent high fractional inspired CO_2_ concentrations to a nonrebreathing mask while maintaining its filtration and isolation capabilities.

Computational Fluid Dynamics (CFD) studies have assessed the CO_2_ distribution in the mask, which showed that CO_2_ was trapped within the mask region.^10–14^ These studies investigated the effect of N95 on CO_2_ levels around the mask region, but the airflow from inside the respiratory airway was excluded. Zhang *et al.*^13^ included the upper respiratory airway, which demonstrated excessive CO_2_ inhalation in every breathing cycle, and that the presence of a respirator may affect the respiratory airflow through nasal breathing. The air-conditioning function of the nasal cavity includes heat and mass exchange between the mucosal wall and airflow,^15,16^ which primarily occurs in the anterior region of the nasal cavity between the nasal valve and the turbinates.^17,18^ Lindermann *et al.*^19^ demonstrated that the mucosal surface temperature varies during different respiration phases. However, previous CFD nasal air-conditioning studies have been performed as a steady-state analysis.^20,21^ During respiration, it is expected that warm exhaled air accumulates within the mask and this is returned to the nasal cavity during inspiration, altering the typical heat and mass exchange between the fresh air and mucosal wall.

Dbouk and Drikakis^22^ investigated the transmission of respiratory droplets through and around a face mask filter using CFD simulations. The results demonstrated that wearing a face mask reduced the droplet travel distance to half, and the mask efficiency varied in different coughing situations. Based on the results of this study, which showed that several droplets could be transmitted meters away from the subject, it was recommended that social distancing is essential during the pandemic. The same authors^23^ proposed a novel three-dimensional multiphase Eulerian–Lagrangian CFD solver to examine the impacts of weather conditions on airborne virus transmission. They also concluded that steady-state relationships induce significant errors and must not be applied in unsteady saliva droplet evaporation.

This study investigated the effects of an N95 respirator on nasal respiration by quantifying the breathing condition during respiration, and its effect on the nasal cavity anatomy and physiology. A human nasal cavity geometry fitted with an N95 respirator was used to explore the respiration flow behavior, and a respiration cycle with a tidal volume of 500 ml was modeled for eight consecutive cycles. A mucosal sub-wall model was applied to allow the analysis of heat and mass transfer between the mucus and inhaled air, thereby producing a net mucosal wall temperature change and humidity changes in the air. The air was treated as a multi-species gas that included water vapor and CO_2_. These species were monitored to track the amounts passing through the nostrils during respiration.

## II. METHOD

### A. CFD model creation

A high-resolution Computed Tomography (CT) scan of a healthy 25-year-old female with no history of previous sinonasal pathology, trauma or surgery, and no anatomical abnormalities was used to create the nasal airway computational model. A Siemens Dual Source CT Scanner (Siemens Healthcare, Erlangen, Germany) was used for the scanning, with the following imaging parameters: 0.39 × 0.39 mm pixel size, 512 × 512 pixel image dimensions, and a slice thickness of 0.6 mm. Before the CT scan, written informed consent was obtained from the subject.

The 3D model reconstruction of the nasal airway from the CT scan was carried out using 3D Slicer^®^ segmentation software. The paranasal sinuses do not affect the nasal airflow significantly;^24,25^ hence, the frontal, maxillary, ethmoid and sphenoid sinuses were removed from the model. To mimic the effects of wearing an N95 respirator, a 3D model of an N95 model was imported into Ansys SpaceClaim^®^, positioned and aligned over the human face. The respirator covered the nostril, and a gap between the N95 mask and human face was considered to represent the natural leakage of a non-fully sealed respirator. Vaseline is usually used to fully seal the N95 mask;^26^ however, this is uncommon and the respirator is not fully sealed most of the time.

### B. Meshing and boundary conditions

**FIG. 1.**
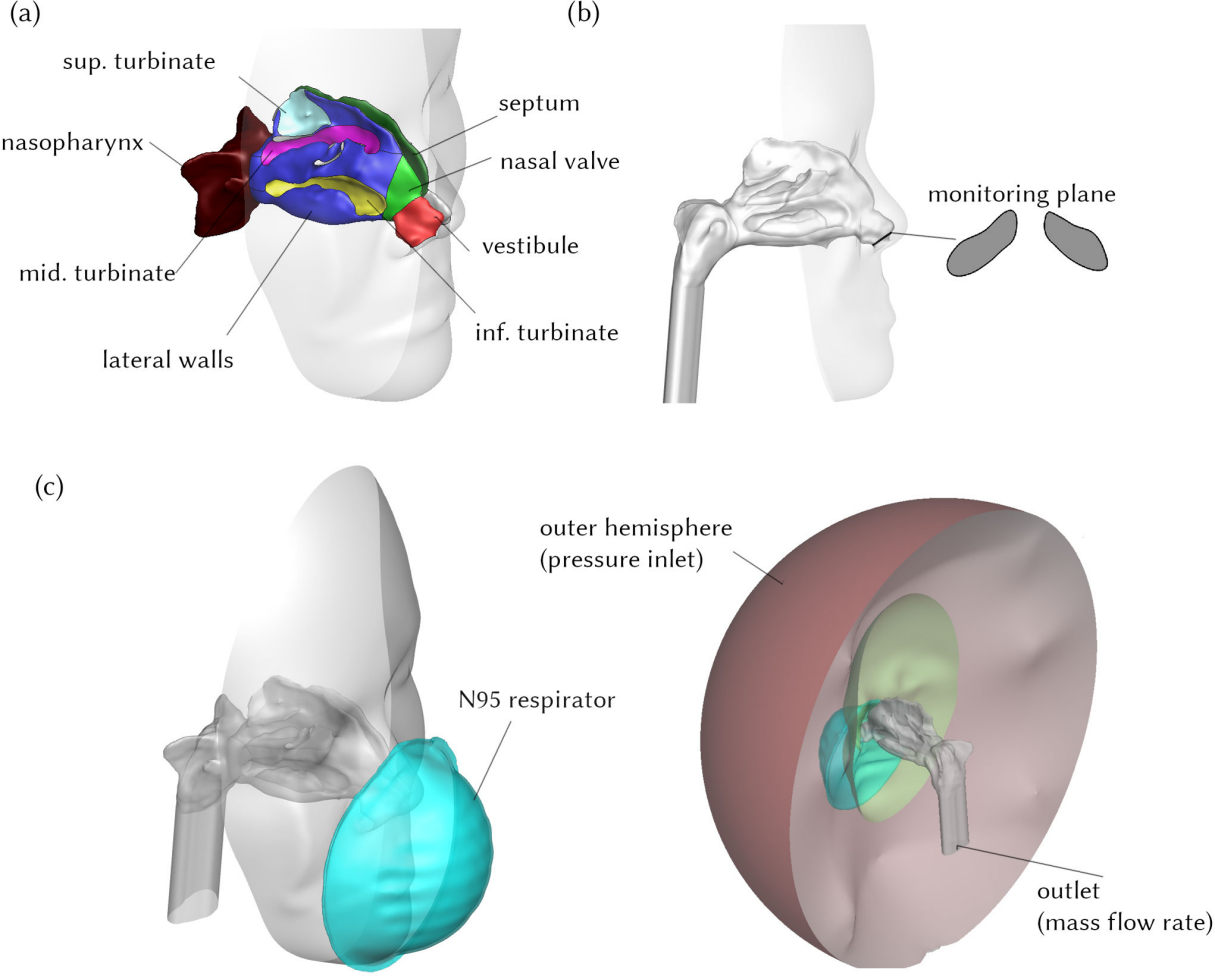
Nasal cavity geometry and computational domain. (a) Anatomical regions of the nasal cavity. (b) Nostrils plane, which is used to monitor the flow properties during both inhalation and exhalation. (c) Computational domain and boundary conditions.

**FIG. 2.**
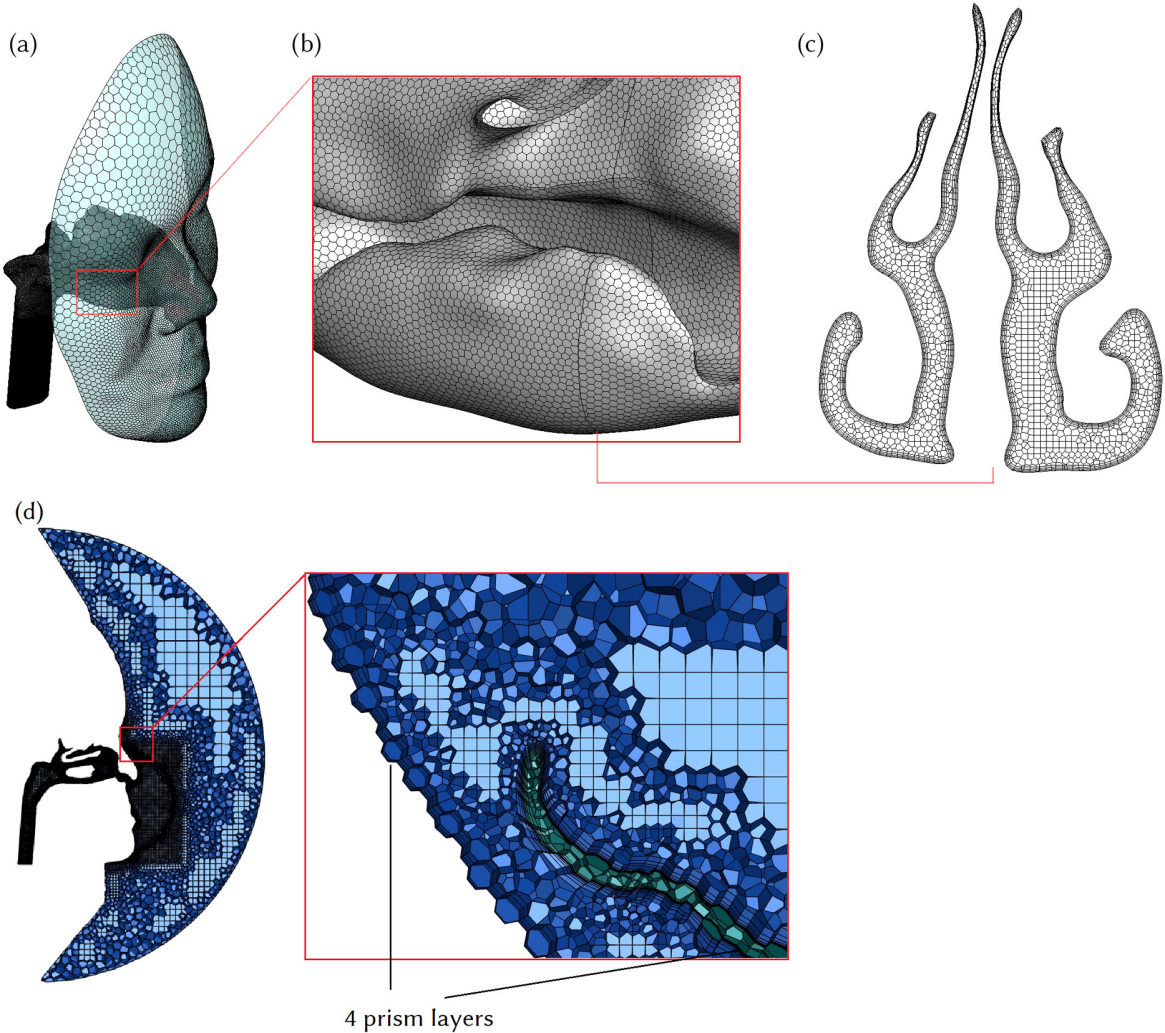
(a) Face and nasal cavity. (b) Zoom view of nasal cavity meshing. (c) Cross-sectional plane in the middle nasal cavity. (d) Sagittal cross-sectional plane in the mid-right nasal passage with a magnified view showing the prism layers used at the respirator and face surfaces.

The geometry of the face was retained and an enclosed hemisphere representing the outer surrounding air (Fig. 1) was constructed in front of the face. A poly-hexcore mesh was generated using Ansys Fluent 2020R2. Mesh independence analysis was performed and checked by plotting velocity contours across the nasopharynx region for three different meshes following Inthavong *et al.*^27^ The final optimized mesh contained 1.5 mil poly-hexcore cells, which contained five prism layers on nasal cavity walls and four prism layers on respirator and face surfaces. This had a total of 6.99 × 10^6^ faces and 5.1 × 10^6^ nodes (Fig. 2), and mesh independence testing results are shown in supplementary material S1. The advantage of poly-hexcore meshing is that it uses fewer elements, approximately 3.5× fewer than tetrahedral meshing with the same size functions. To accurately capture the boundary layer profile, five prism layers with a first-layer thickness of 0.06 mm were used in the nasal cavity. Four prism layers were used on each side of the respirator surface (interior and exterior) and face surface to capture the flow complexities through and around the mask [Fig. 2(d)]. A body of influence with sizing of 0.8 mm was used around the nasal cavity and respirator to create a local mesh refinement and avoid a larger mesh size in these regions.

The exterior surface of the domain was set to atmospheric pressure, and respiration was initiated by setting a defined mass flow rate at the exit of nasopharynx extension [Fig. 1(c)]. The respiratory cycle was simplified to pure sine waves based on the measured physiology data from Benchetrit *et al.*^28^ and used in Calmet *et al.,*^29^ allowing a simple method for describing the tidal volume, breathing periods, and periodicity, e.g.,

$$\dot{m}=A\;\text{sin}\;\left(\frac{\pi (t-C)}{B}\right).$$ (1)

For inhalation, the amplitude $A=5.832\times 10^{-4}$ kg/s and period *B* = 1.65 s, while the periodicity for multiple breathing cycles is C=4(n−1) s, where *n* is the respiration cycle number. For exhalation, $A=4.0945\times 10^{-4}$ kg/s; *B* = 2.35 s; and C=(1.65+4(n−1)) s. This produces a tidal volume of 500 ml, where the inhalation period is 1.65 s and the exhalation period is 2.35 s. The solution was initialized at time *t* = 0 s with steady-state settings, and therefore, we exclude the first inhalation phase to avoid startup effects from the analysis. This ensures that the respiration results represent continuous breathing.

Simulations for breathing without a respirator were performed for two respiration cycles, while breathing with a respirator was carried out for eight respiration cycles to investigate the cumulative impact of the respirator on the inhaled airflow. The first four cycles used a time step of $\Delta t=0.25\times 10^{-3}$ s, taking the simulation time to 17.65 s. The final four cycles were modeled for evaluating the inhaled gas mixtures only. Larger time steps were used based on their ability to predict similar values as the original time step, evaluated over the peak inhalation period between 12.5 and 13 s.

**FIG. 3.**
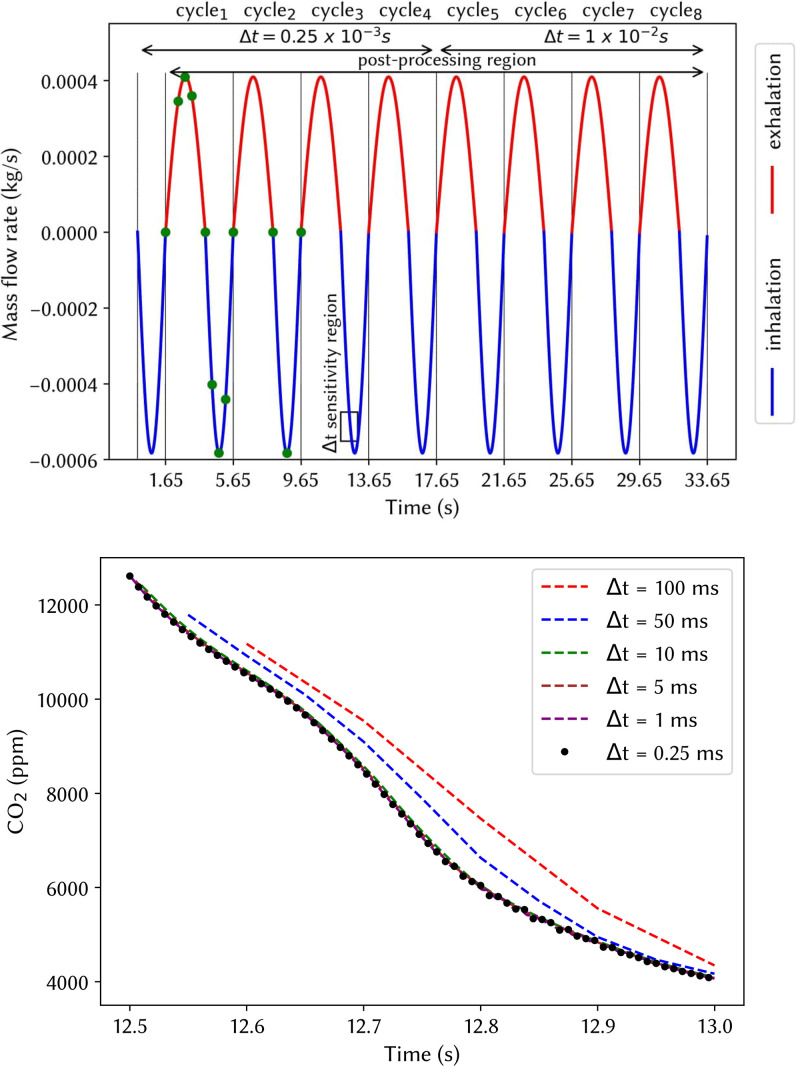
(a) Respiration cycle mass flow rate profile assigned to the nasopharyngeal. (b) Time step sensitivity analysis. Average CO_2_ concentration passing through the monitoring plane for different Δt (ms).

Figure 3(a) shows the assigned flow respiratory profile with the first four cycles modeled with $\Delta t=0.25\times 10^{-3}$ s, and the last four cycles modeled with $\Delta t=1\times 10^{-2}$ s, where Fig. 3(b) shows that the latter time step size was the most efficient time step while maintaining the same results. These time-steps are consistent with previous respirator CFD studies^10–14^ that used $\Delta t=5\times 10^{-2}$ s to perform transient respirator breathing simulations.

Monitored points of interest were selected at the beginning, peak, and end of inspiration and expiration cycles. Additional points before and after peak inspiration and expiration were included to demonstrate the acceleration and deceleration around the peak. Equal time intervals of 0.4 s between each point were selected.

The mask zone was assumed as a porous medium with a porosity of 0.88, and a viscous resistance coefficient of $1.12\times 10^{10}$ m^−2^ based on Zhang *et al.*^13^ The filtration material of the N95 respirator is a poly-propylene fabric that has a low thermal conductivity, which varies between 0.11 and 0.22 W/m K as reported by Patti *et al.*^30^ Thus, N95 mask walls were considered adiabatic. A plane was created at the nostril opening and oriented normal to the flow to monitor the gas mixtures entering and existing the nasal cavity during respiration [Fig. 1(b)].

### C. Numerical setup

Airflow modeling was performed assuming a transient, laminar flow through the nasal airway, mimicking a full breath cycle using the commercial CFD code, Ansys Fluent 2020R2 (ANSYS Inc., Canonsburg, PA, US). Laminar flow characteristics were found to be dominant for flows at 15 l/min.^31^ While some turbulence will occur at peak flows of approximately 30 l/min at the nasopharynx, the effect is expected to be small. The impingement of the air jet onto the mask surface can be categorized based on the critical Reynolds number, as suggested by Gardon *et al.*^32^ We considered the maximum velocity at the nostril and the nostril diameter, which gives a Reynolds number less than 1000, which is classified as a laminar jet.

The ambient air temperature was set to 20 °C, while that of the exhaled air was set to 36 °C. The exhaled air was assumed to be fully saturated and has a CO_2_ mass fraction of 36 000 ppm.^33^ The ambient air relative humidity and CO_2_ mass fraction were 30% and 385 ppm, respectively.^33^ The governing equations can be expressed in the form of the following transport equation:

$$\;\frac{\partial \rho \phi }{\partial t}+\nabla \cdot (\rho \Phi v)=\nabla (\Gamma_{\phi }\nabla \phi )+S_{\phi }.$$ (2)

The generalized scalar ϕ, diffusion coefficient $\Gamma_{\phi }$, and source term $S_{\phi }$ for each governing equation are defined in Table I.

**TABLE I.** Summary of governing equations.

Equation	ϕ	$S_{\phi }$	$\Gamma_{\phi }$
continuity	1	0	0
*x* momentum	U	$-\frac{\partial p}{\partial x}+\frac{\partial }{\partial x}(\mu \frac{\partial U}{\partial x})+\frac{\partial }{\partial y}(\mu \frac{\partial U}{\partial x})+\frac{\partial }{\partial z}(\mu \frac{\partial U}{\partial x})$	*μ*
*y* momentum	V	$-\frac{\partial p}{\partial y}+\frac{\partial }{\partial x}(\mu \frac{\partial V}{\partial y})+\frac{\partial }{\partial y}(\mu \frac{\partial V}{\partial y})+\frac{\partial }{\partial z}(\mu \frac{\partial V}{\partial y})$	*μ*
*z* momentum	W	$-\frac{\partial p}{\partial z}+\frac{\partial }{\partial x}(\mu \frac{\partial W}{\partial z})+\frac{\partial }{\partial y}(\mu \frac{\partial W}{\partial z})+\frac{\partial }{\partial z}(\mu \frac{\partial W}{\partial z})$	*μ*
Energy	T	$\frac{\partial }{\partial x}(\sum_{i}h_{i}J_{i})+\frac{\partial }{\partial y}(\sum_{i}h_{i}J_{i})+\frac{\partial }{\partial z}(\sum_{i}h_{i}J_{i})$	$\frac{\mu }{\rho Pr}$
Species mass fraction	*Y_i_*	0	$\rho D_{i}$

Here, *μ* is laminar viscosity (kg/m.s), *T* is temperature (K), *C_p_* is specific heat capacity (J/kg.K), Pr is laminar Prandtl number, *h_i_* is the specific enthalpy of species *i*, **J**_*i*_ is the diffusive flux of species *i*, *Y_i_* is species mass fraction, and *D_i_* is the diffusion coefficient for species *i* in the mixture (m^2^/s).

The nasal cavity wall boundary was set with a virtual thickness with length *L* to represent the mucus and submucosal layers (supplementary material S2) that contain epithelial cells and a bed of blood vessels that provide a heat source. An effective thermal resistance, R=L/kA, was applied, where *A* is the area normal to the conduction direction. The remaining conductivity and thickness parameters were defined following Na *et al.*^34^ who reviewed anatomic information on the respiratory mucosa from Beule *et al.*^35^ The effective resistance used was R=0.020 K/W. The temperature along the base of the nasal cavity wall boundary was set to $36{\;}^{{}^{\circ}}$C representing core-body temperatures, except for the nasal vestibule which was set to 34 °C,^17,19,36,37^ slightly lower based on its external location to the internal nasal cavity. The nasal vestibule in humans is completely lined by stratified squamous epithelium where its primary function is to protect the underlying layers. No humidity exchange occurs in this region, and we expect a reduced heat transfer exchange through the subepithelium to provide the temperature source. In contrast, the nasal mucosa epithelium from the nasal valve to the nasopharynx exhibits a dense subepithelial network of capillaries that generate an underlying body core temperature of $37{\;}^{{}^{\circ}}$C.

The nasal cavity walls were assumed to be wet and to have a saturated state due to a layer of mucus on the surface. The water-vapor mass fraction is at the surface boundary was set assuming a 100% relative humidity condition. Since the surface boundary temperature varies with the flow conditions an approximate function relating the saturated water-vapor concentration with temperature was created giving the following equation:

$$C_{\text{surface}}=\frac{1}{1000}(0.0006312T^{3}-0.01097^{2}+0.6036T+2.027),$$ (3)

where $C_{\text{surface}}$ is the water vapor concentration in kg m^−3^ and *T* is the temperature in °C. The current nasal wall model has been previously used by Senanayake *et al.*^38^ in the analysis of nasal airflow and mucosal heat and mass transfer.

To obtain the solution, the second order upwind discretization scheme was used for momentum, energy, species, and the pressure–velocity coupling used the SIMPLE scheme. A second-order implicit scheme was used for the temporal discretization of the transient term.

## III. RESULTS

**FIG. 4.**
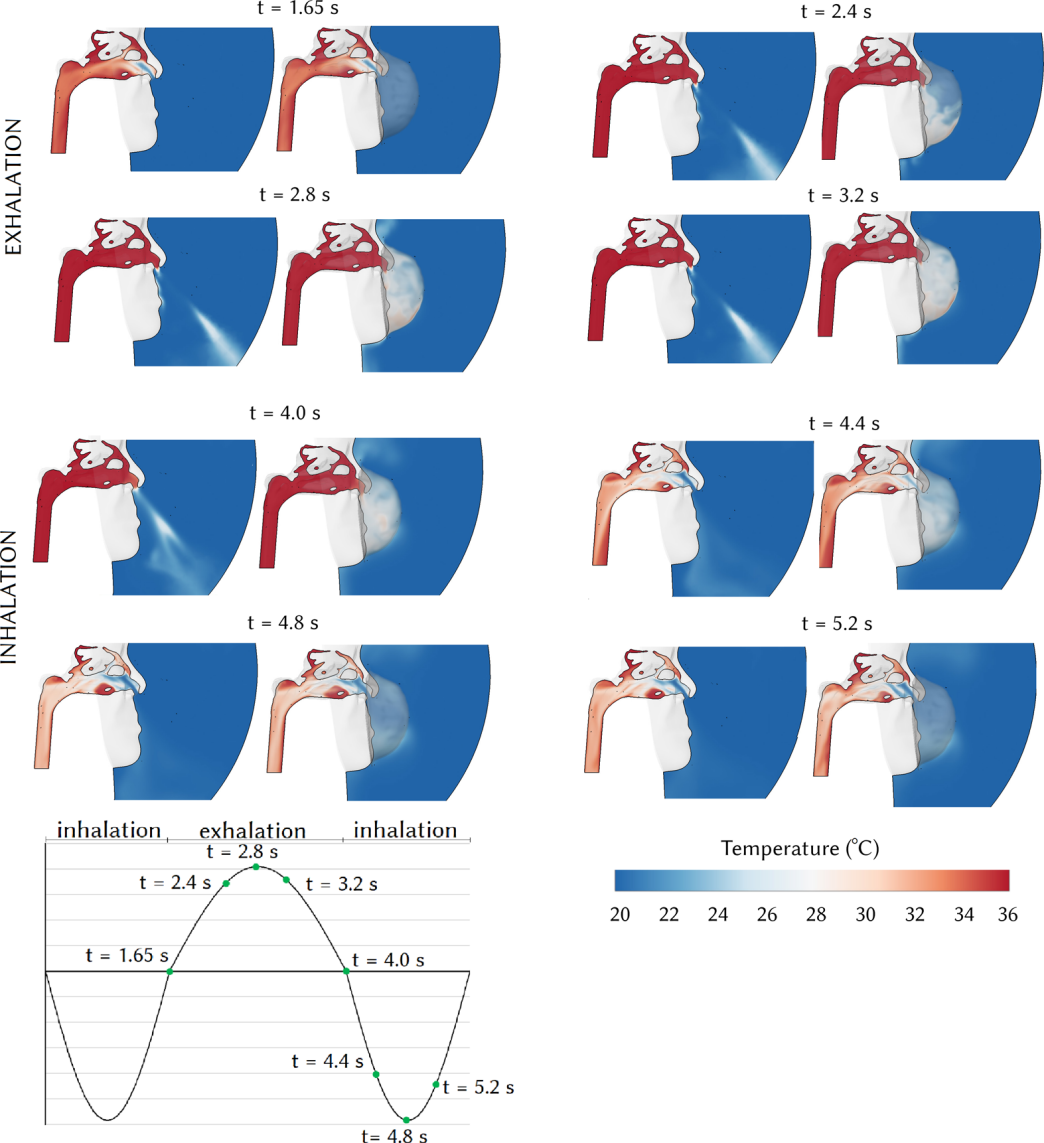
Air temperature distribution at the mid-sagittal plane of the right nasal passage with and without a respirator during exhalation-inhalation cycle between 1.65 and 5.65 s.

The air temperature field taken at the mid-sagittal plane through the right nasal cavity with and without a mask is shown for different times during the first respiratory cycle in Fig. 4. During exhalation without a respirator, the air temperature in the nasal cavity recovers to its maximum of 36 °C. The warm air exits the nostrils and mixes with the cold surrounding environment air. When inhalation begins, colder ambient air enters the nasal cavity where heat transfer takes place between the air and heated mucus wall. The air is initially cold at the anterior region of the nasal cavity and is warmed as it moves toward the posterior region. During respirator breathing, the high-temperature exhaled air exits the nostrils and accumulates in the mask and the temperature inside the respirator increases during exhalation (*t* = 1.65 to *t* = 4.0 s). During inhalation, the accumulated high-temperature air in the respirator enters the nasal cavity, and the temperature in the respirator decreases. The temperature comparison within the nasal cavity between normal and respirator breathing at *t* = 4.4 s shows that the airflow temperature for respirator breathing is higher. The nasal airflow differences reduce at *t* = 5.2 s when the mass flow rate decreases.

**FIG. 5.**
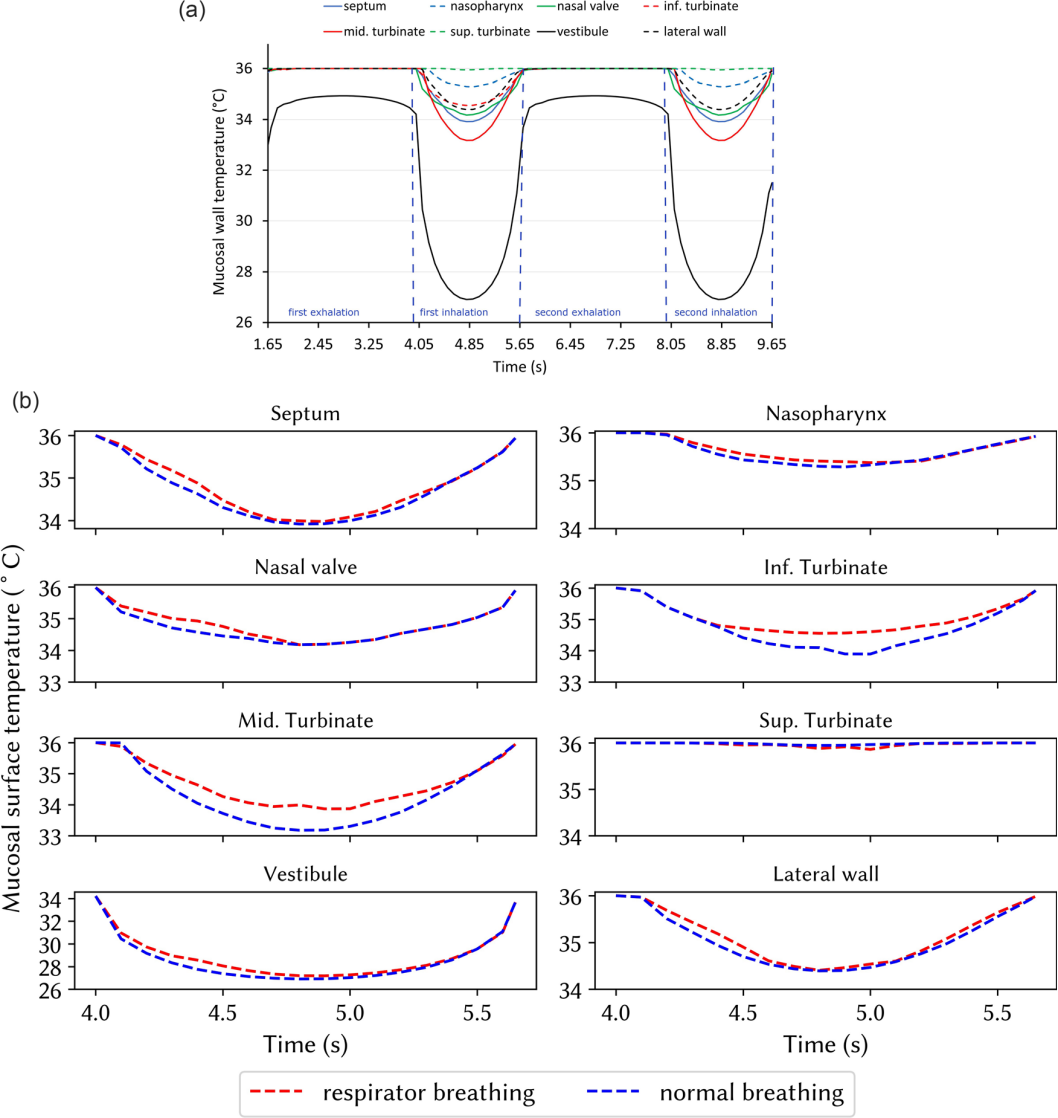
(a) Mucosal surface temperature variation during two respiration cycles without a respirator. (b) Mucosal surface temperature variation during the first inhalation with and without a respirator.

Figure 5(a) illustrates the baseline results (without a respirator) of the mucosal wall temperature at different locations during two respiration cycles. In all regions except the vestibule, the exhaled temperature remained the same at $36{\;}^{{}^{\circ}}$ C, where no heat transfer occurred between the exhaled breath and mucosal walls because of the same temperature and saturation state. At the beginning of the inhalation, the mucosal wall temperature decreased due to the outer air temperature of $20{\;}^{{}^{\circ}}$ C and reached minimum values at peak inhalation before returning to the initial temperature at the end of the breath cycle. During peak inhalation, the mucosal surface temperature at the middle turbinate and septum reached 33 °C and 34 °C, respectively.

Figure 5(b) compares individual mucosal wall temperatures with and without a respirator during the inhalation period, 4.0 s <t< 5.65 s. Each mucosal surface temperature decreased to a minimum at peak inhalation before returning to its original temperature at the end of inhalation. Temperatures in the anterior nose were cooler than the posterior regions, as the inhaled air warmed as it moved posteriorly. The mucosal surface temperature of the superior turbinate was 36 °C for both respirator and normal breathing caused by a reduced airflow around this region. The middle turbinate surface temperature increased to 34 °C for respirator breathing, which was a 1 °C increase compared with normal breathing.

**FIG. 6.**
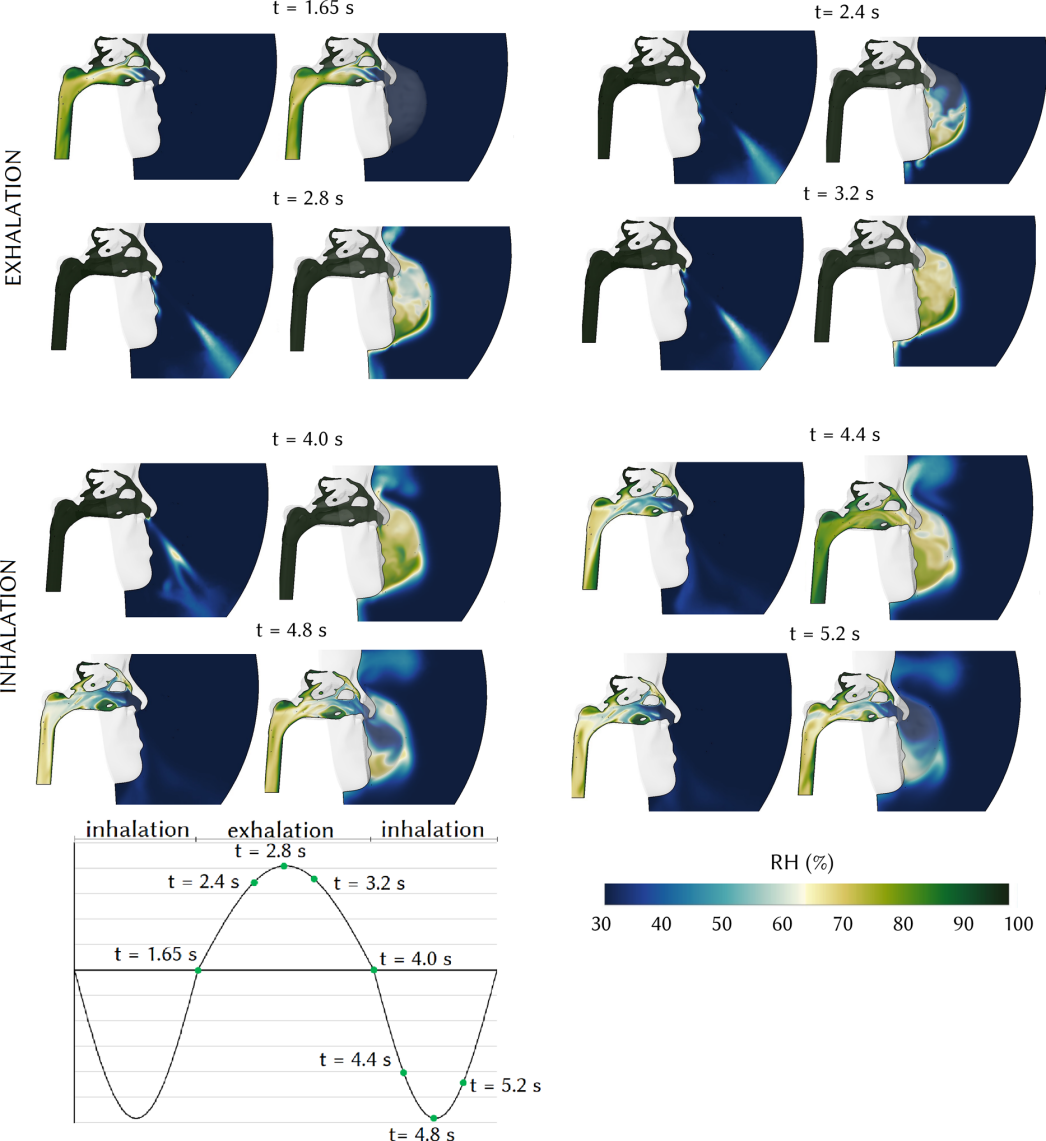
Relative humidity at the mid-sagittal plane of the right nasal passage without a respirator.

The water vapor mass fraction distribution, shown as relative humidity, RH (Fig. 6), in the mid-sagittal plane of the right nasal passage is similar to the temperature distribution. The airflow coming from the lung during exhalation is 100% humid and similar for normal and respirator breathing. During normal breathing, the humid exhaled air exits the nostrils and mixes with the ambient air. When inhalation begins, the ambient air with RH of 30% enters the nasal cavity. The presence of an N95 respirator traps the humid exhaled air and RH level within the respirator increases from *t* = 2.4 to *t* = 4.0 s. The N95 respirator increased the relative humidity of the inhaled air especially at *t* = 4.4 and *t* = 4.8 s. The RH within the respirator reached 90% at *t* = 4 s at the end of expiration. During inspiration, the water vapor diffusion from the mucosal surface reduced due to inhaling more humid air from the respirator.

**FIG. 7.**
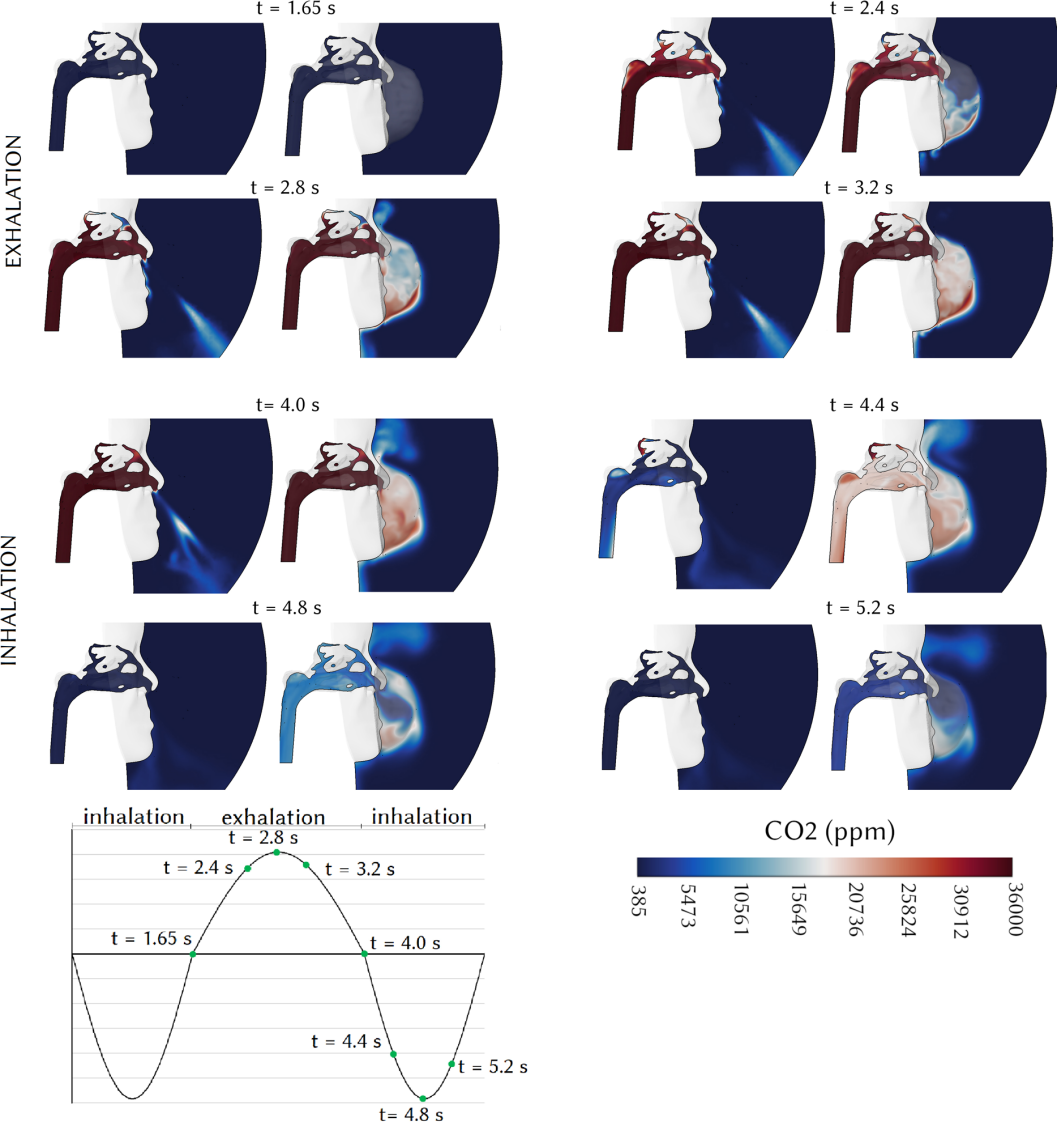
CO_2_ distribution at the mid-sagittal plane of the right nasal passage with and without a respirator.

**FIG. 8.**
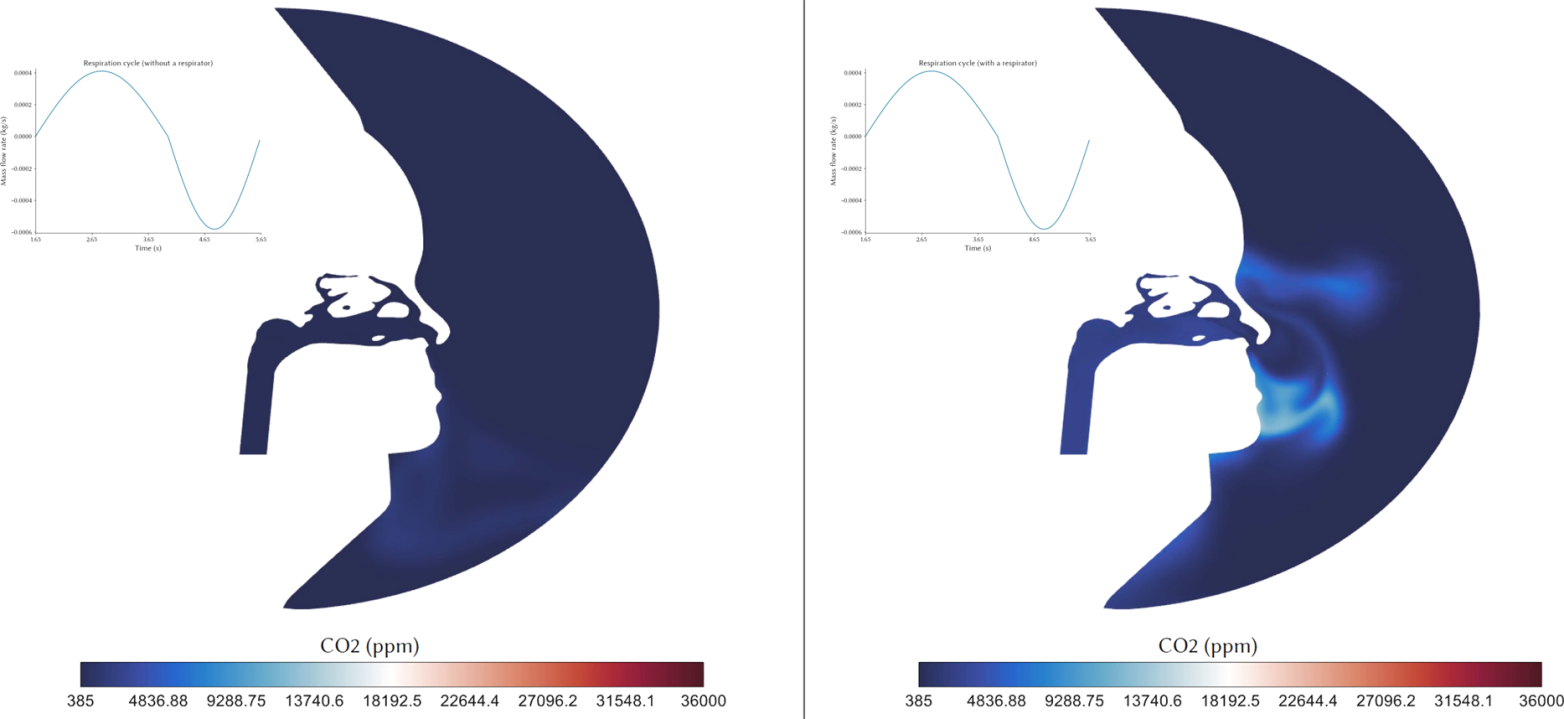
Animation of CO_2_ distribution at the mid-sagittal plane of the right nasal passage with and without a respirator. Multimedia view: https://doi.org/10.1063/5.0061574.1
10.1063/5.0061574.1

The CO_2_ distribution in the mid-sagittal plane of the right nasal passage during the first respiration cycle is shown in Fig. 7 with and without a respirator. In both cases, the CO_2_ distribution is high during exhalation as it is discharged from the lungs moving toward the nostrils. After *t* = 3.2 s, the CO_2_ is distributed to the superior regions and fills the nasal cavity. Inhalation without mask provided fresh air and the CO_2_ levels in the nasal cavity diminished (from *t* = 4.4 to 5.65 s). At *t* = 4.4 s, the average CO_2_ concentration entering the nasal cavity was 575 ppm, which was close to the ambient level (Fig. 7), suggesting the absence of any CO_2_ augmentation. The animation of CO_2_ distribution at the mid-sagittal plane of the right nasal passage with and without a respirator is shown in Fig. 8 (Multimedia view). During breathing with a respirator mask, the exhaled air with the CO_2_ level of 36 000 ppm exited the nostrils and accumulated within the respirator region. The CO_2_ level increased to 3200 ppm within the mask at *t* = 4.0 s. This was 5.5× greater than the CO_2_ level of fresh air breathing. Consequently, there was a significant difference in the inhaled CO_2_ concentrations between breathing with and without a respirator mask. The multimedia view showed in both breathing cases that accumulated CO_2_ in the nasal cavity was transported away from inhalation, and into the lungs, and therefore, a larger proportion of the exhaled air is returned to the lungs.

**FIG. 9.**
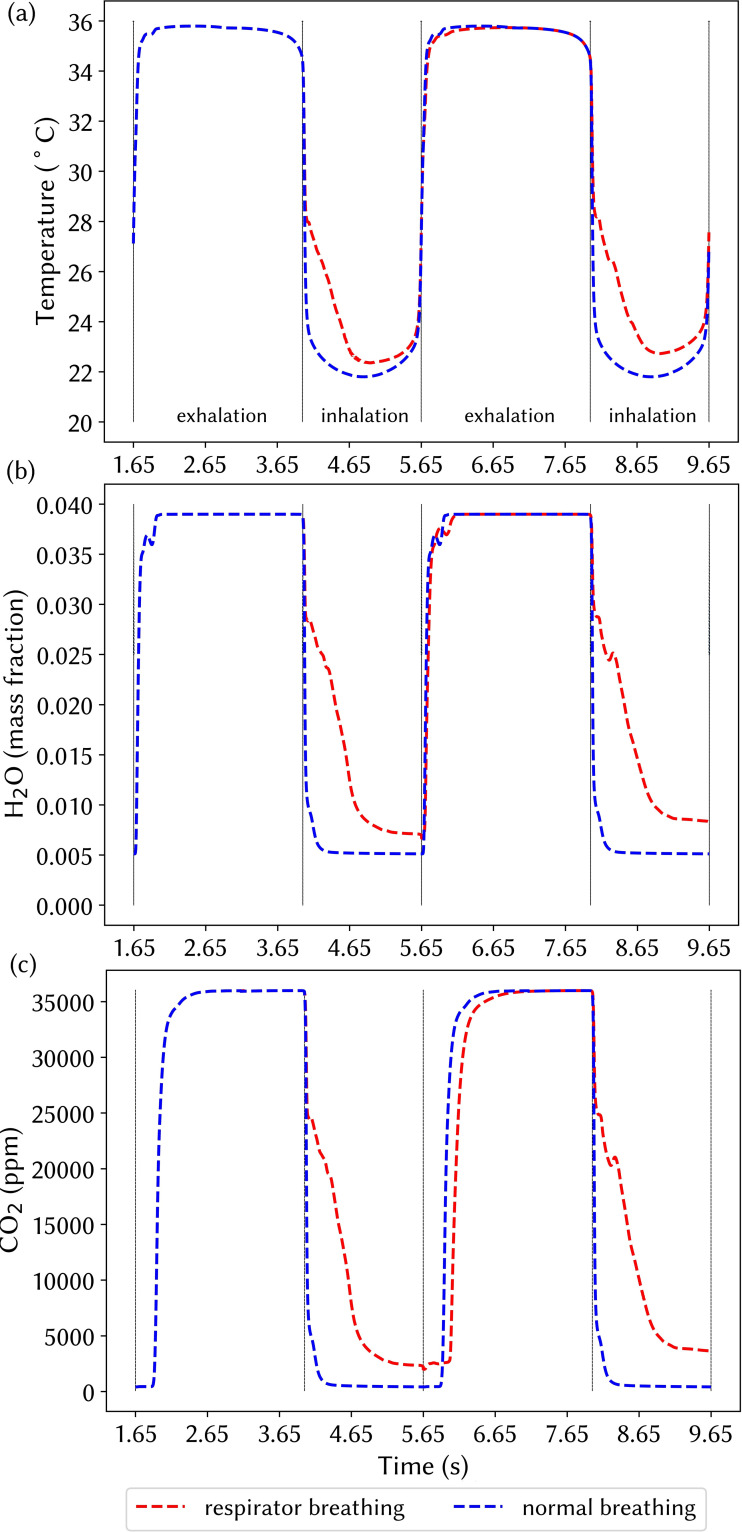
(a) Temperature, (b) water-vapor mass fraction, and (c) CO_2_ concentration passing through the monitoring plane for the cases with a respirator and normal breathing.

Figure 9 presents the temperature, water-vapor content, and CO_2_ concentration exiting (exhalation) and entering (inhalation) the nasal cavity through the nostril openings, monitored over two respiration cycles with and without a respirator. Breathing with a respirator increased the uptake of all parameters, i.e., increase in the moisture and CO_2_ content and temperature. The gas mixtures inhaled increased slightly during the second cycle compared with the first cycle for the respirator breathing. At *t* = 4.4 s, the averaged inhaled air temperature was 25.2 °C, while without a respirator, it was 22.2 °C. The inhaled air temperature difference was more notable during the first half of the inhalation compared with the second half. The temperature, H_2_O, and CO_2_ concentration values differ between normal and respirator breathing. The time average of each variable, denoted by ϕ during the inhalation, was obtained by

$$\bar{\phi }=\frac{1}{1.65}\int_{t_{i}}^{t_{i}+1.65}\phi \left(t\right)dt,\;$$ (4)

where *t_i_* is the start of the inhalation period at different respiration cycles. The time-averaged air temperature during an inhalation period for normal breathing was $T_{\mathrm{ave}}=22.59$ °C. The effect of a respiratory mask caused an increase in the temperature to $T_{\mathrm{ave}}=24.03$ °C (6% difference). In the second inhalation cycle, the normal breathing conditions produced the same inhaled temperature profile, while the temperature value for the mask breathing increased to $T_{\mathrm{ave}}=24.41$ °C (8% difference). Similarly, the time-averaged H_2_O mass fraction entering the nasal cavity was 0.0060 for normal breathing, and this increased to 0.0139 (131% difference) and 0.0150 (150% difference) for the first and second cycles during respirator breathing, respectively. During inhalation at each respiration cycle, the CO_2_ content entering the nasal cavity started from 36 000 ppm, and at the end of inhalation, it reached 3000 ppm. The average inhaled CO_2_ for inhalation without a respirator was 1445 ppm, while this value was 9472 ppm (555% difference) and 10,555 ppm (630% difference) for respirator breathing at the first and second inhalation, respectively.

The residual exhaled air inside the mask builds up consecutively with each breathing cycle, hence increasing the air temperature, H_2_O, and CO_2_ concentrations during inhalation. A ratio of the time-averaged values of the air properties over each inhalation cycle for respirator mask breathing to the constant normal breathing conditions is defined as follows:

$$\phi_{\text{ratio}}=\frac{{\bar{\phi }}_{\text{respirator}\;\text{breathing}}}{{\bar{\phi }}_{\text{normal}\;\text{breathing}}}.$$ (5)

**FIG. 10.**
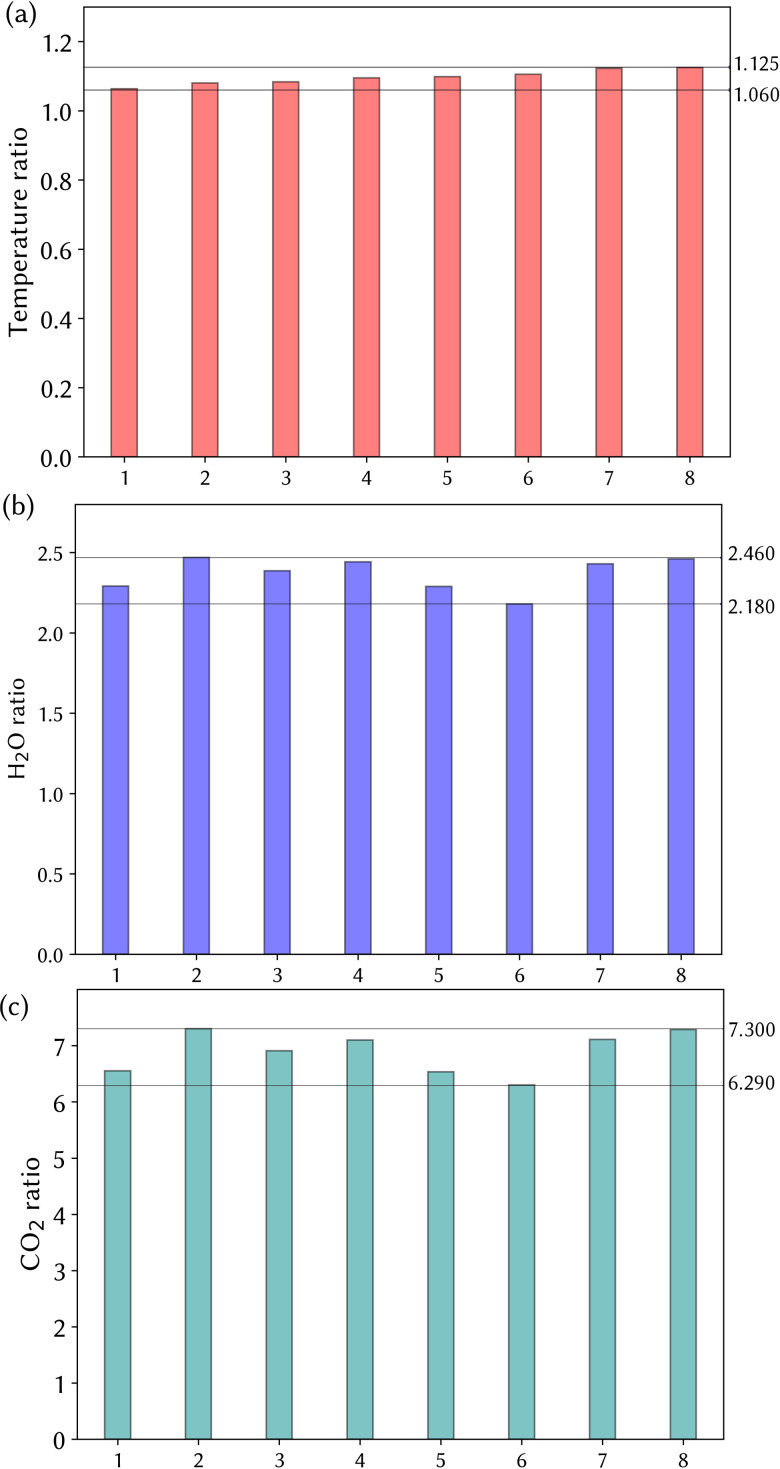
The ratio value for (a) temperature, (b) H_2_O concentration, and (c) CO_2_ concentration. The ratio of each variable shows the time-average of the variable with a respirator present over that without a respirator present for each respiration cycle.

Thus, $\phi_{\text{ratio}}$ represents the fractional change in the respirator mask breathing to normal breathing for each during each inhalation breath over the eight cycles that was modeled. A $\phi_{\text{ratio}}=1$ represents no change. Figure 10 demonstrates these results where the temperature ratio consistently but gradually increased from 1.06 (respiration cycle 1) to 1.13 (respiration cycle 8). The H_2_O mass fraction oscillated between 2.18 and 2.46. The CO_2_ concentration ratio varied from 6.3 to 7.3.

## IV. DISCUSSION

Respiration with the N95 respirator showed the exhaled air exiting the nostrils accumulate in the mask and is unable to mix with the surrounding air, causing an increase in the air temperature inside the mask. Consequently, the inhaled air is at a higher temperature than the ambient air, leading to a decreased heat exchange between the mucosal wall and the inhaled air. The reduced cooling effect was most significant in the middle and inferior turbinates where the main flow paths move through, leading to higher surface temperatures than what would occur during normal breathing. The activation of trigeminal “cool” thermoreceptors during nasal mucosal cooling is known to be the first mechanism that produces the sensation and perception ample nasal airflow.^39–41^ The inhibiting effect of the respirator on the heat and mass exchange may exacerbate the sensation of achieving sufficient breathing physiologically and psychologically.

The respirator mask restricts the ability of the exhaled air to mix with the ambient air, leading to an accumulation of CO_2_ inside the mask. At the end of exhalation, CO_2_ concentration reached 28 000 ppm, whereupon instead of inhaling fresh air containing a low CO_2_ concentration, the accumulated CO_2_ in the mask is inhaled. The stale exhaled air inside the mask is transferred back into the nasal cavity and into the lungs. At the end of inhalation, the CO_2_ concentration in the mask is replenished by the exhaled breath, but reaches a limit where excessive amounts escape through the gaps in the mask attachment to the face. Exposure to a CO_2_ concentration of 10 000 ppm for 30 min or more in a healthy adult results in respiratory acidosis. Furthermore, excessive CO_2_ can cause increased respiratory rate, metabolic stress, increased brain blood flow, and increased minute ventilation.

Breathing with a respirator mask led to an increase in inhaled CO_2_ by 6.3 to 7.3 times per breath. This high CO2 concentration build up inside the mask was relatively constant during respiratory breathing, as minor leakages around the respirator regulated further build-up. For fully sealed masks with higher efficiencies aimed at suppressing aerosol penetration, the CO_2_ concentration level build-up would be expected to increase further. Exposure to high CO_2_ concentrations, e.g., >30 000 ppm results in headaches, dizziness, and dyspnea.^42^ Therefore, users of high efficient masks without CO_2_ regulation should reduce the amount of time they are wearing the mask. While leakages around the mask increase the aerosol penetration exposure, it does reduce high CO_2_ exposure. A further investigation of the effect of mask seal on the aerosol penetration into the mask, and aerosol dispersion from sneezing or coughing could be useful. The CO_2_ tracking demonstrated that the exhaled airflow from the nostrils impinges onto the bottom of the respirator, and CO_2_ accumulation starts in this region. This suggests that fans and valves can be placed in this region to immediately replace high CO_2_ content-air with fresh ambient air.

The respirator seal with the face can be reduced by different parameters, such as a beard or poorly fitted to the facile anatomy. The seal influences the amount of exhaled air exchange with the ambient air and its impact on inhalation. Future work can be performed on the effect of face mask seal on the leakage parameter and the nasal airflow. The results are sensitive to different breathing and ambient conditions, including temperature, humidity, and breathing flow rate, and its effect on the accumulation of the exhaled air in the mask could be further investigated. Nevertheless, this study quantified the increase in temperature, H_2_O, and CO_2_ concentration levels in human nasal cavity anatomy breathing with a respirator.

## V. CONCLUSION

CFD simulations were performed over eight respiration cycles for normal and respirator breathing. The nasal airflow and temperature were compared between the two different simulations. The assessed variables for normal breathing were consistent with each breath cycle. For respirator breathing, the mucosal wall temperature increased compared with normal breathing. The breathing flow field with and without a respirator showed that the N95 respirator significantly affected the flow properties, including CO_2_ concentration, temperature, and humidity of the inhaled air.

The effect of breathing through a respirator has increased the time-averaged inhaled air temperature by 8%; H_2_O mass fraction by 150%; and CO_2_ concentration by 555% during the first inhalation cycle. Throughout eight breaths, the maximum change as a ratio to normal breathing was temperature increased by 1.13 times; H_2_O mass fraction increased by 2.46 times; and CO_2_ concentration increased by 7.3 times. The increase in the inhaled temperature and humidity decreased the cooling effect on the mucosal surface, which is thought to affect the physiological sensation of full breathing. Furthermore, the continual inhalation of excessive CO_2_ concentrations can lead to detrimental health effects on the subject.

The current findings and limitations of this study inspire future work for respirator breathing research. A comprehensive parametric study can be performed to identify the parameters, which alter the breathing condition due to wearing a respirator. These parameters can potentially be respirator sealing, environmental temperature and humidity, indoor ventilation conditions (downward or upward ventilation), outdoor ambient conditions (wind velocity and direction), and human motion.

## SUPPLEMENTARY MATERIAL

See the supplementary material for the mesh independence results and a figure of the submucosal wall model.

## Data Availability

The data that support the findings of this study are available from the corresponding author upon reasonable request.
